# TCA-croton oil peels: unpredictable behavior and risk of severe hypertrophic scarring^☆^

**DOI:** 10.1016/j.abd.2026.501455

**Published:** 2026-08-28

**Authors:** Carolina Reato Marçon, Seaver L. Soon, Carlos Gustavo Wambier

**Affiliations:** a Dermatology Department, Santa Casa de Misericórdia de São Paulo, São Paulo, SP, Brazil; b The Skin Clinic MD (private practice), San Diego, CA, United States; c Department of Dermatology and Cutaneous Surgery, Miller School of Medicine, University of Miami, Miami, FL, United States; d Department of Dermatology, Warren Alpert Medical School, Brown University, Providence, RI, United States

Dear Editor,

Following the temporary ban on medical use of phenol in Brazil in 2024,^1^ alternative deep peel formulations rapidly emerged on social media in an attempt to replace the well-established phenol-croton oil peel, particularly compounded Trichloroacetic Acid (TCA)-croton oil combinations. In a porcine model, Lemes et al. demonstrated that a 35% TCA-croton oil formulation induced significantly less inflammation and resulted in a more superficial depth of injury compared with Hetter’s phenol-croton oil formulation, suggesting important physicochemical and biological differences between these compounds.^2^ In this context, we report a series of patients presenting with cicatricial complications after facial peels using 35% TCA combined with croton oil.

This report includes patients evaluated in our practice for cicatricial complications following TCA-croton oil peels. Among 10 consecutive patients treated by the authors with this formulation, 3 developed severe hypertrophic scarring (30% incidence). In addition, 2 patients referred by other physicians were evaluated and managed for similar complications, resulting in a total of 5 patients included in the analysis. All patients were female (age range, 32–84 years) and had no known pre-existing risk factors for hypertrophic scarring, such as prior keloids, connective tissue disease, or impaired wound healing. The procedures were performed for the treatment of photoaging and acne scars. Given the descriptive nature of this report, no formal statistical analysis was performed. All patients provided written informed consent for publication of their clinical data and images. This study was conducted in accordance with the principles of the Declaration of Helsinki.

Clinically, hypertrophic scars were characterized by marked erythema, dermal thickening, irregular surface, and firm adherence to deeper planes, suggesting deep dermal and subcutaneous injury. The perioral and maxillary regions were most frequently affected, with associated functional impairment, including restricted oral opening and ectropion (Fig. 1). While some patients exhibited outcomes resembling those historically observed with classical phenol-croton oil peels, others developed extensive and functionally significant scarring, highlighting the apparent unpredictability of TCA-croton oil formulations (Fig. 2). Due to the high incidence and severity of complications, the authors discontinued the use of this formulation.

**Fig. 1:**
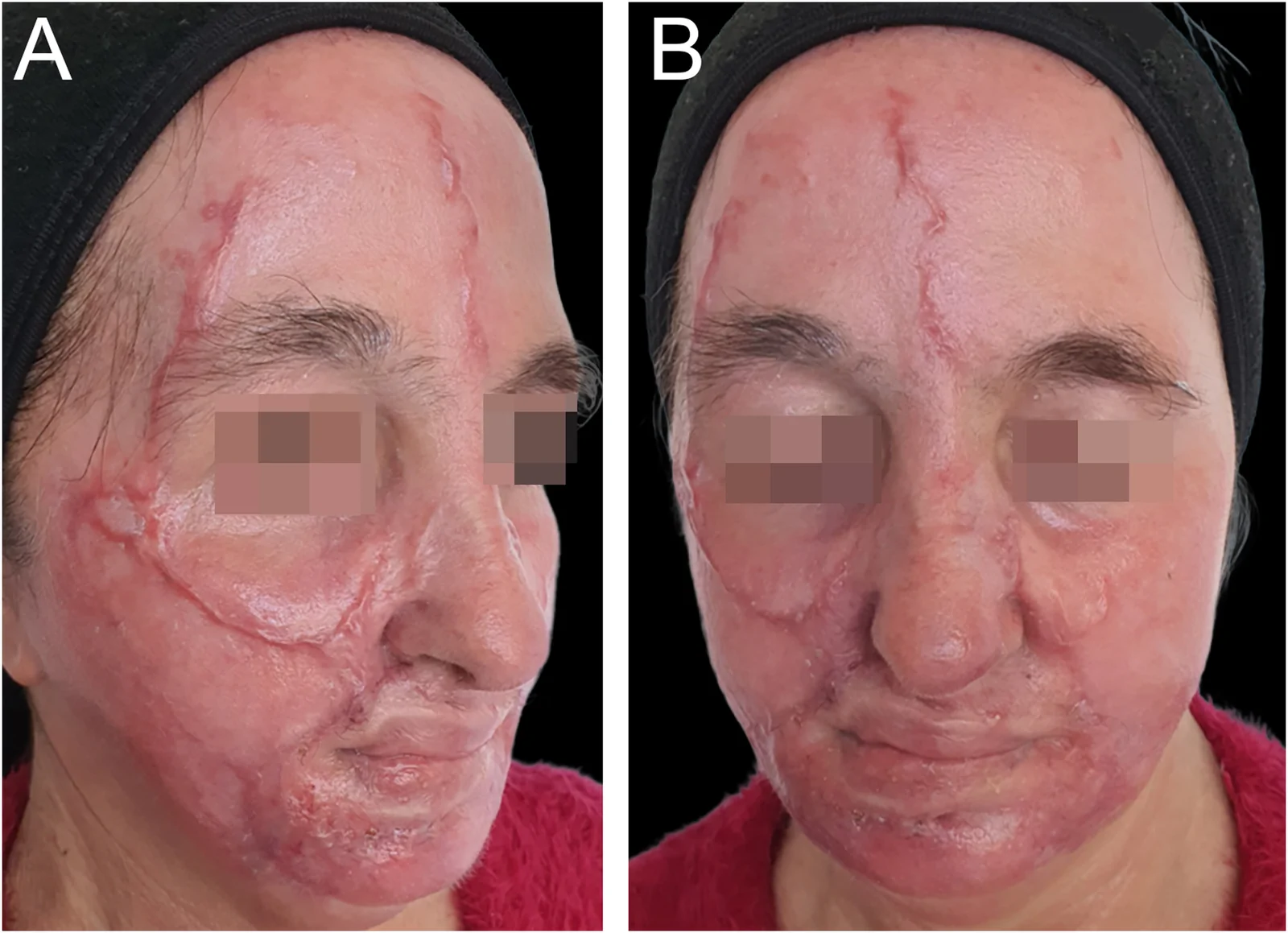
Hypertrophic scarring following TCA-croton oil peel with a linear and geographic distribution pattern. (A) Right oblique view showing well-demarcated erythematous hypertrophic scars along the forehead, periorbital, malar, and perioral regions. (B) Frontal view highlighting the symmetric distribution of lesions, with prominent perioral involvement and areas of crusting and incomplete re-epithelialization.

**Fig. 2:**
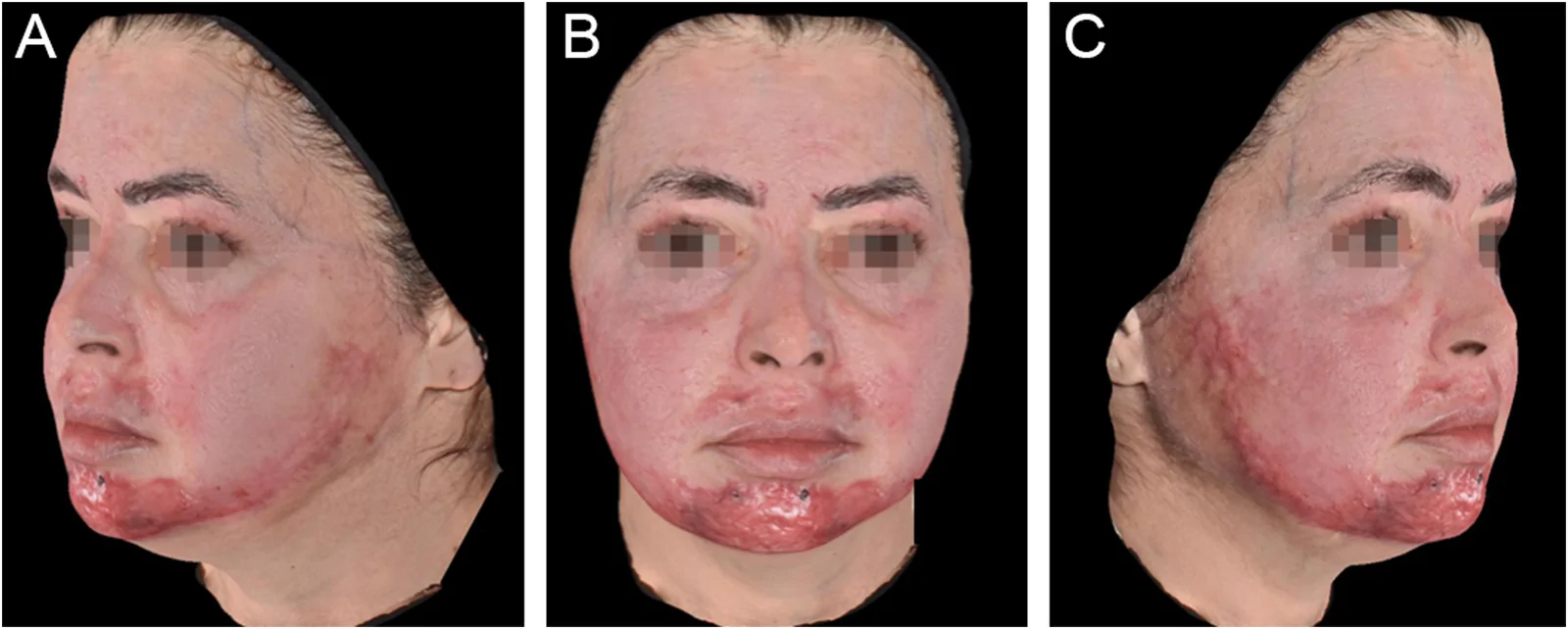
Severe hypertrophic scarring following TCA-croton oil peel. (A) Left lateral view showing marked erythema, dermal thickening, and involvement of the mandibular and perioral regions. (B) Frontal view demonstrating diffuse facial erythema with prominent perioral fibrosis. (C) Right lateral view highlighting extensive scarring affecting the malar and perioral areas, with evident tissue contraction.

Trichloroacetic acid at 35% concentration is traditionally associated with superficial-to-medium depth chemical peeling and a relatively low risk of scarring.^3^, ^4^ However, factors such as overcoating and excessive volume may increase penetration depth due to its strong protein-coagulating and caustic properties.^4^ When combined with croton oil, whose phorbol esters are potent activators of protein kinase C, the formulation may induce intense neutrophilic inflammation, including Neutrophil Extracellular Trap (NET) formation,^5^ as well as deeper follicular penetration and coagulation. Additionally, TCA is a hydrophilic acid, whereas phenol is lipophilic and acts as a solvent for croton oil, suggesting fundamentally different penetration dynamics. The resulting clinical effects may resemble those observed with high-concentration TCA (>50%), historically associated with a higher risk of scarring.^4^

Based on these observations, TCA-croton oil formulations appear to exhibit unpredictable behavior and a clinically significant risk of severe scarring, even in patients without identifiable risk factors. Until further in vitro studies and controlled clinical trials establish their safety and reproducibility, their use should be approached with extreme caution and should not be considered equivalent to established phenol-croton oil formulations.

## Authors’ contributions

Carolina R. Marçon: Study conception; data collection, analysis, and manuscript writing.

Seaver L. Soon: critical revision of the manuscript and intellectual contribution.

Carlos Gustavo Wambier: Critical revision of the manuscript, data analysis, and manuscript writing.

## Financial support

None declared.

## Research data availability

Does not apply.

## Conflicts of interest

Carlos Gustavo Wambier serves as an advisor for Young Pharmaceuticals, Inc and is an inventor in the USPTO patent #11,253,465 on methods of emulsification of phenol-croton oil formulas.
